# Initiating and imaging cavitation from infused echo contrast agents through the EkoSonic catheter

**DOI:** 10.1038/s41598-023-33164-5

**Published:** 2023-04-16

**Authors:** Sonya R. Kennedy, Maxime Lafond, Kevin J. Haworth, Daniel Suarez Escudero, Dan Ionascu, Brion Frierson, Shaoling Huang, Melvin E. Klegerman, Tao Peng, David D. McPherson, Curtis Genstler, Christy K. Holland

**Affiliations:** 1 Department of Internal Medicine, Division of Cardiovascular Health and Disease, University of Cincinnati, Cardiovascular Center 3935, 231 Albert Sabin Way, Cincinnati, OH 45267-0586 USA; 2 Department of Biomedical Engineering, University of Cincinnati, Cincinnati, OH USA; 3 Department of Radiation Oncology, College of Medicine, University of Cincinnati, Cincinnati, OH USA; 4 Department of Internal Medicine, The University of Texas Health Science Center at Houston, Houston, TX USA; 5 Boston Scientific, Maple Grove, MN USA; 6 Present Address: LabTAU, Inserm, Université Lyon 1, Lyon, France

**Keywords:** Cardiac device therapy, Interventional cardiology, Biomedical engineering, Cardiovascular diseases

## Abstract

Ultrasound-enhanced delivery of therapeutic-loaded echogenic liposomes is under development for vascular applications using the EkoSonic Endovascular System. In this study, fibrin-targeted echogenic liposomes loaded with an anti-inflammatory agent were characterized before and after infusion through an EkoSonic catheter. Cavitation activity was nucleated by Definity or fibrin-targeted, drug-loaded echogenic liposomes infused and insonified with EkoSonic catheters. Passive cavitation imaging was used to quantify and map bubble activity in a flow phantom mimicking porcine arterial flow. Cavitation was sustained during 3-min infusions of Definity or echogenic liposomes along the distal 6 cm treatment zone of the catheter. Though the EkoSonic catheter was not designed specifically for cavitation nucleation, infusion of drug-loaded echogenic liposomes can be employed to trigger and sustain bubble activity for enhanced intravascular drug delivery.

## Introduction

Peripheral arterial disease (PAD) represents a challenging clinical problem affecting 15–20% of people over 70 years of age due to the diffuse nature of atheroma deposition throughout the arterial bed^1^. Management of PAD with angioplasty and bare metal stents is complicated by restenosis. The use of drug eluting stents in PAD has reported disappointing long-term results^2^. Innovative strategies that prevent the buildup of additional plaque in the intervention area, reduce inflammation in the surrounding atheroma bed, and promote healthy blood flow may improve clinical outcomes.

**Figure 1:**
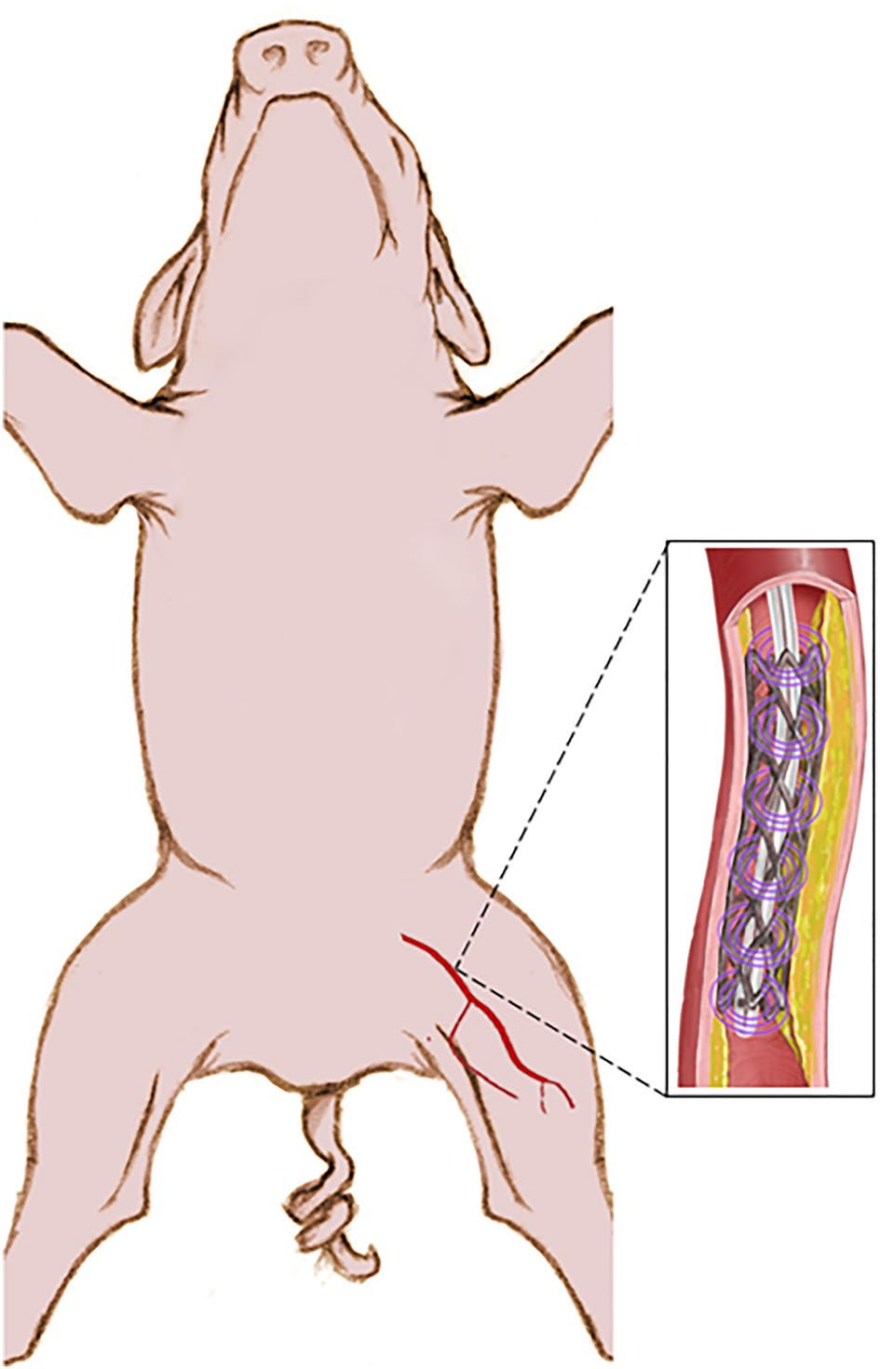
Concept for ultrasound-medicated delivery of pioglitazone-loaded echogenic liposomes infused through an EkoSonic catheter into the porcine iliofemoral arterial vasculature.

One such strategy is the delivery of agents to stabilize the atheroma in the peri-stent area^3^. Echogenic liposomes (ELIP) are lipid-bilayer agents that encompass a gas-filled monolayered microbubble which renders the particles sonosensitive^4,5^. Sensitivity to ultrasound (US) enables ELIP to be visible on US images. Furthermore, ELIP can be triggered by US for the release and delivery of therapeutics^3,5–9^. Octafluoropropane (OFP) can be used to stabilize ELIP against dissolution due to the low solubility of OFP in aqueous solutions, including blood^4,10^. ELIP can be functionalized by coating the shell surface with drugs or targeting agents^3,11^. As microbubbles oscillate in the vasculature, these effects can open endothelial tight junctions^12–14^, induce microstreaming^15^, and increase drug transport across the endothelium^16,17^. Pioglitazone (PGN), an anti-inflammatory drug, has been shown to limit inflammation in vasculature^18–20^. Therapeutic delivery has been demonstrated in a porcine model with resultant atheroma stabilization using this strategy (Fig. 1)^9^.

ELIP loaded with pioglitazone and targeted to fibrin with a custom-made nonapeptide, OFP-PAFb-PGN-ELIP, are under development to provide an adjunct to stents following percutaneous intervention in arteries^3,21^ to stabilize atheroma. The EkoSonic Endovascular System (Boston Scientific, Maple Grove, MN, USA) is an FDA-cleared catheter for ultrasound-mediated infusion of therapeutics into the peripheral vasculature and pulmonary arteries. Lafond et al. demonstrated that Definity infused through the EkoSonic catheter could be used to nucleate sustained cavitation over 3 min.^22,23^ Due to the nucleation of inertial and stable cavitation from infused contrast agents and the potential for deleterious tissue side effects, the FDA requires careful characterization of the cavitation spawned by OFP-PAFb-PGN-ELIP infused through and insonified by the EkoSonic catheter before clinical trials can commence.

The purpose of this study was to determine if infused OFP-PAFb-PGN-ELIP can nucleate sustained cavitation with the EkoSonic catheter. The size distribution, frequency-dependent acoustic attenuation, and PGN content in each vial of ELIP was determined before and after infusion through the EkoSonic catheter. Passive cavitation imaging (PCI) along the distal six active transducer pairs of the EkoSonic catheter was performed throughout 3 min infusions of Definity or OFP-PAFb-PGN-ELIP. Both stable and inertial cavitation activity was quantified and mapped over the range of EkoSonic electrical drive powers (4 to 47 W) used in the FDA-approved clinical pulsed ultrasound protocol.

## Results

### Characterization of OFP-PAFb-PGN-ELIP

**Figure 2:**
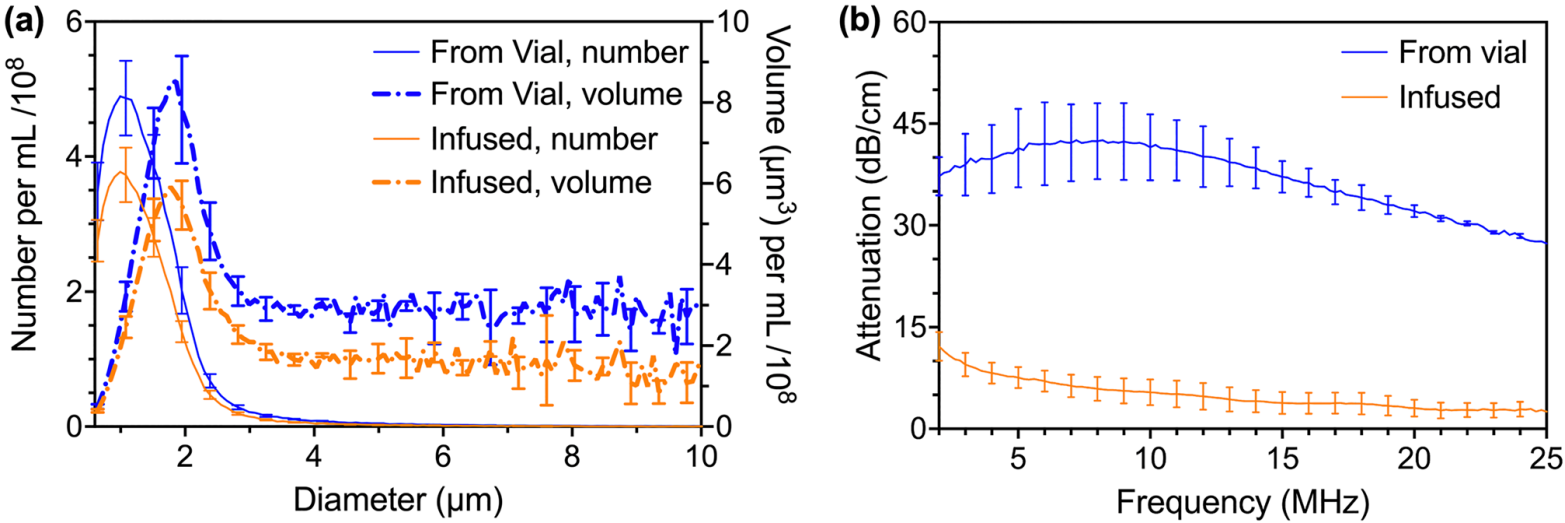
(**a**) Size distribution and (**b**) attenuation spectroscopy measurements of OFP-PAFb-PGN-ELIP from the vial and after infusion through EkoSonic catheters. The mean number-weighted (solid lines) and volume-weighted (dot-dot-dash lines) size distributions are plotted against the particle diameter. The mean attenuation values are plotted as a function of frequency. Error bars represent ± 1 standard deviation (s.d.), *n* = 3 vials each.

Figure 2 depicts the (a) number- and volume-weighted size distribution and (b) ultrasound attenuation as a function of frequency for the reconstituted OFP-PAFb-PGN-ELIP pipetted directly from the vial and after infusion through quiescent 135 cm EkoSonic catheters. Measured directly from the vial, 98.5 ± 0.3% of the OFP-PAFb-PGN-ELIP had a diameter less than five microns. After infusion through the quiescent catheter, 98.9 ± 0.1% were less than five microns. Before infusion, the peak number-weighted concentration of 5.0 × 10^8^ ± 0.6 × 10^8^ microbubbles per mL from the vial (mean ± standard deviation, s.d., *n* = 3) corresponded to a diameter of 1.0 µm. The peak number-weighted concentration after infusion decreased to 3.8 × 10^8^ ± 0.5 × 10^8^ microbubbles per mL (mean ± s.d., *n* = 3) and the modal diameter was 1.0 µm. The peak volume-weighted number density of OFP-PAFb-PGN-ELIP occurred at a diameter of 1.8 µm and the peak decreased from 8.5 × 10^8^ ± 1.4 × 10^8^ μm^3^ per mL from the vial to 5.9 × 10^8^ ± 0.6 × 10^8^ μm^3^ per mL after infusion (mean ± s.d., *n* = 3 each). Two-tailed Welch’s t tests were used to compare the number-weighted (alpha = 0.05, *p* = 0.076) and volume-weighted peak density (alpha = 0.05, *p* = 0.064). The reconstituted OFP-PAFb-PGN-ELIP exhibited 30 to 40 dB attenuation over a range of frequencies from 2 to 25 MHz. The center frequency of the EkoSonic transducer pairs is 2.25 MHz. The attenuation of reconstituted OFP-PAFb-PGN-ELIP at 2.2 MHz decreased from 37.8 ± 4.4 dB/cm to 11.4 ± 1.9 dB/cm after infusion. OFP-PAFb-PGN-ELIP from the vial exhibited peak attenuation at resonant frequencies in the 5.0 to 10.0 MHz range and infused OFP-PAFb-PGN-ELIP exhibited peak attenuation at 1.6 to 2.2 MHz (maximum ± 1 dB/cm, *n* = 3 each).

### Measurement of pioglitazone dose

**Figure 3:**
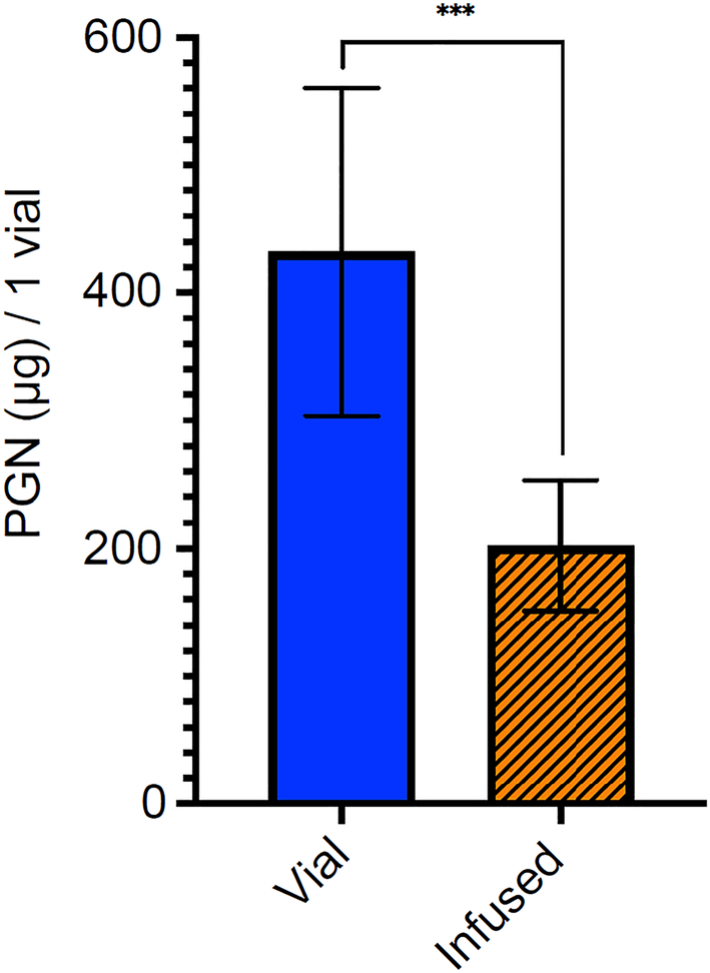
Mean PGN dose of one vial of reconstituted OFP-PAFb-PGN-ELIP measured directly from the vial and after infusion through EkoSonic catheters. Each vial contains 0.5 mL reconstituted solution and 10 mg lipid/ mL solution. Error bars represent ± s.d., *n* = 3 vials each.

Using high-performance liquid chromatography (HPLC), the PGN dose was measured directly from three reconstituted vials of OFP-PAFb-PGN-ELIP and an additional three vials after infusion through the EkoSonic catheter. Figure 3 depicts the PGN doses of 432.0 ± 128.3 µg and 202.0 ± 51.2 µg (mean ± s.d., *n* = 3 each), quantified directly from the vial and after infusion through quiescent EkoSonic catheters, respectively. With an intra-class correlation of 0.50, the PGN doses directly from the vial and after infusion through the catheter were correlated necessitating that correlation should be considered to examine their differences. The difference is significant (alpha = 0.05, *p* = 0.0007).

### Spatial light interference microscopy of Definity and OFP-PAFb-PGN-ELIP

**Figure 4:**
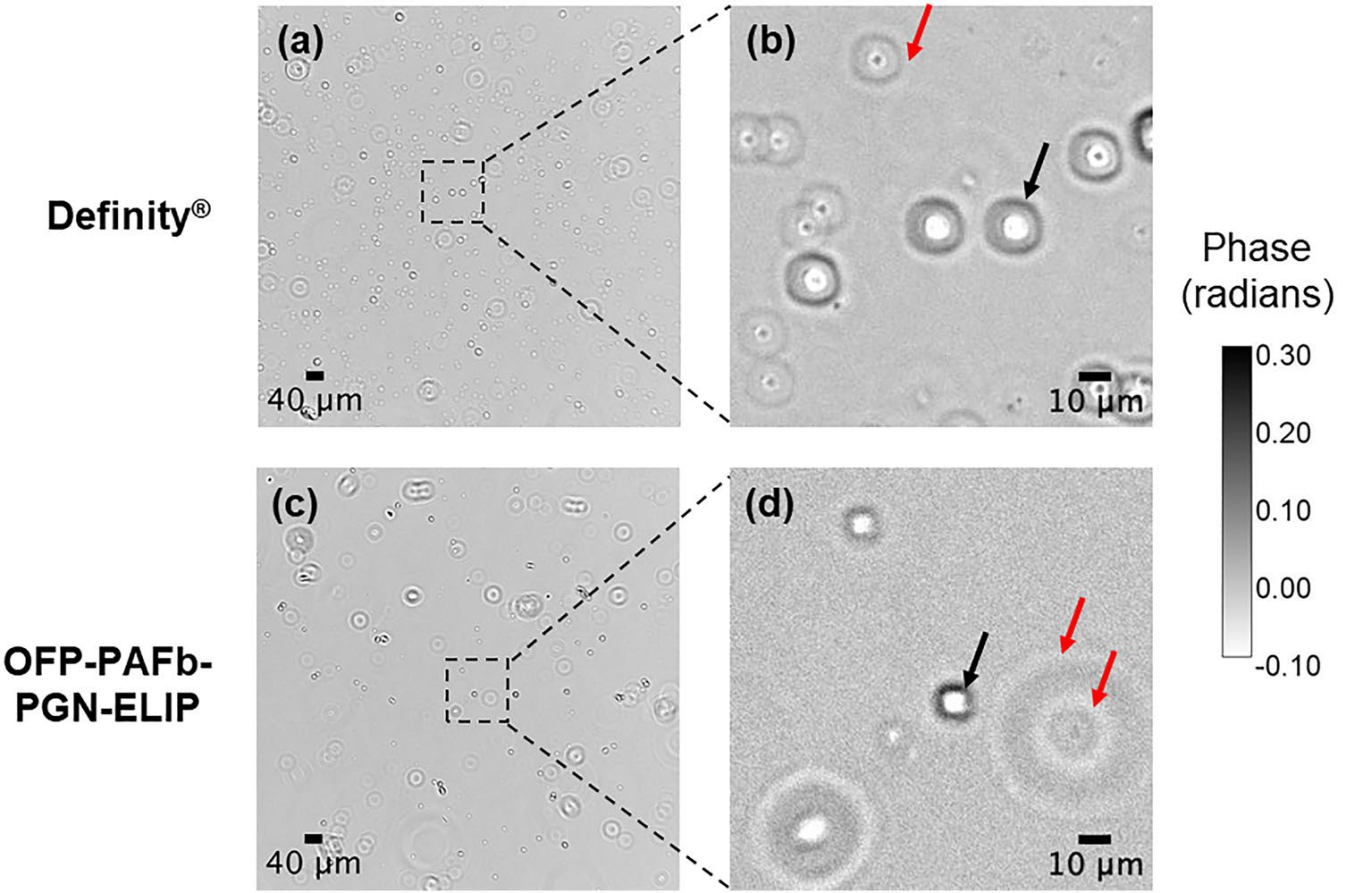
Spatial light interference microscopy (SLIM) images of **(a, b)** Definity and **(c, d)** OFP-PAFb-PGN-ELIP using a 10 × objective. Images were taken of microbubbles withdrawn directly from vials (no infusion). The black arrows indicate lipid shells, and the red arrows indicate diffraction artifacts which appear as white rings.

Spatial light interference microscopy (SLIM) of Definity and OFP-PAFb-PGN-ELIP is shown in Fig. 4. White represents gas cores, and black represents the lipid coating of the particles the imaging plane. The spherical nature of the lipid-shelled microbubbles is revealed with z-stack acquisitions (Supplemental Figs. S3 and S4 online). As the microscope imaging plane moves through each particle, the structure of the lipid shell and gas core is observed. The exterior phase ring around the particles is a diffraction artifact.

### Cavitation activity sustained by the EkoSonic catheter in flow

**Figure 5:**
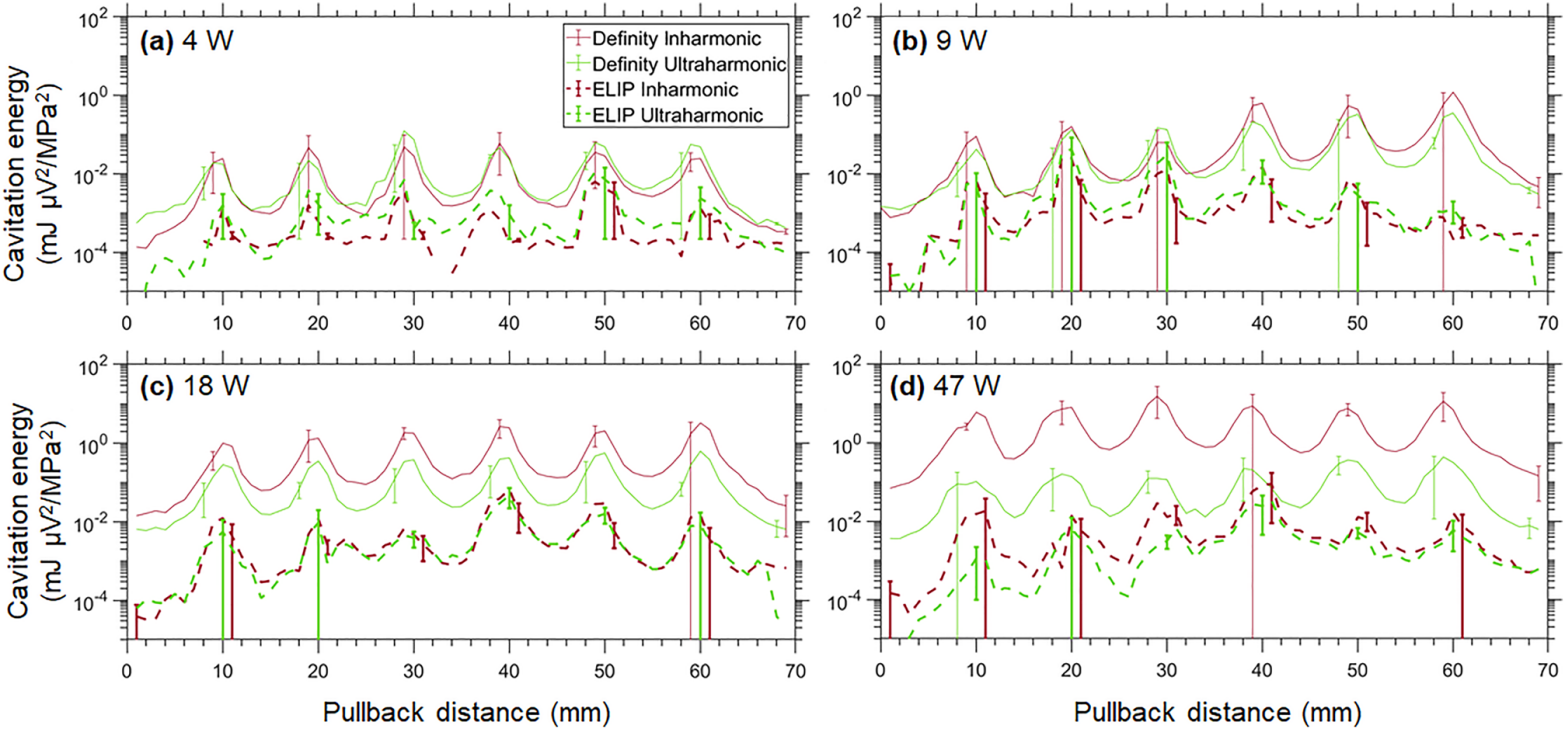
Cavitation energy along the active distal six transducer pairs of the EkoSonic catheter as OFP-PAFb-PGN-ELIP or Definity was infused. The mean ultraharmonic (green) and inharmonic (red) cavitation with electrical drive powers of (**a**) 4, (**b**) 9, (**c**) 18, and (**d**) 47 W. Note that the PCI post-processing protocol plots the ultraharmonic energy in excess of the inharmonic energy. Error bars represent ± 1 standard deviation,* n* = 3 each.

Plotted in Fig. 5 are the ultraharmonic and inharmonic cavitation energy nucleated by Definity or OFP-PAFb-PGN-ELIP infused through the EkoSonic catheter driven at electric powers between 4 and 47 W (*n* = 3 each). Each local maximum corresponds to alignment of the passive L11-5v array with one of the six active transducer pairs in the EkoSonic catheter during pullback. Ultraharmonic and inharmonic cavitation emissions were sustained at all electrical drive powers. For Definity infusions at the 18 and 47 W drive powers, the inharmonic cavitation energy exceeded ultraharmonic cavitation energy. Also, for Definity infusions, the inharmonic cavitation energy increased with electrical drive power. However, for OFP-PAFb-PGN-ELIP infusions at drive powers above 9 W, ultraharmonic and inharmonic energies were similar. Overall, Definity nucleated one to two orders of magnitude more cavitation energy than OFP-PAFb-PGN-ELIP at equivalent drive powers.

**Figure 6:**
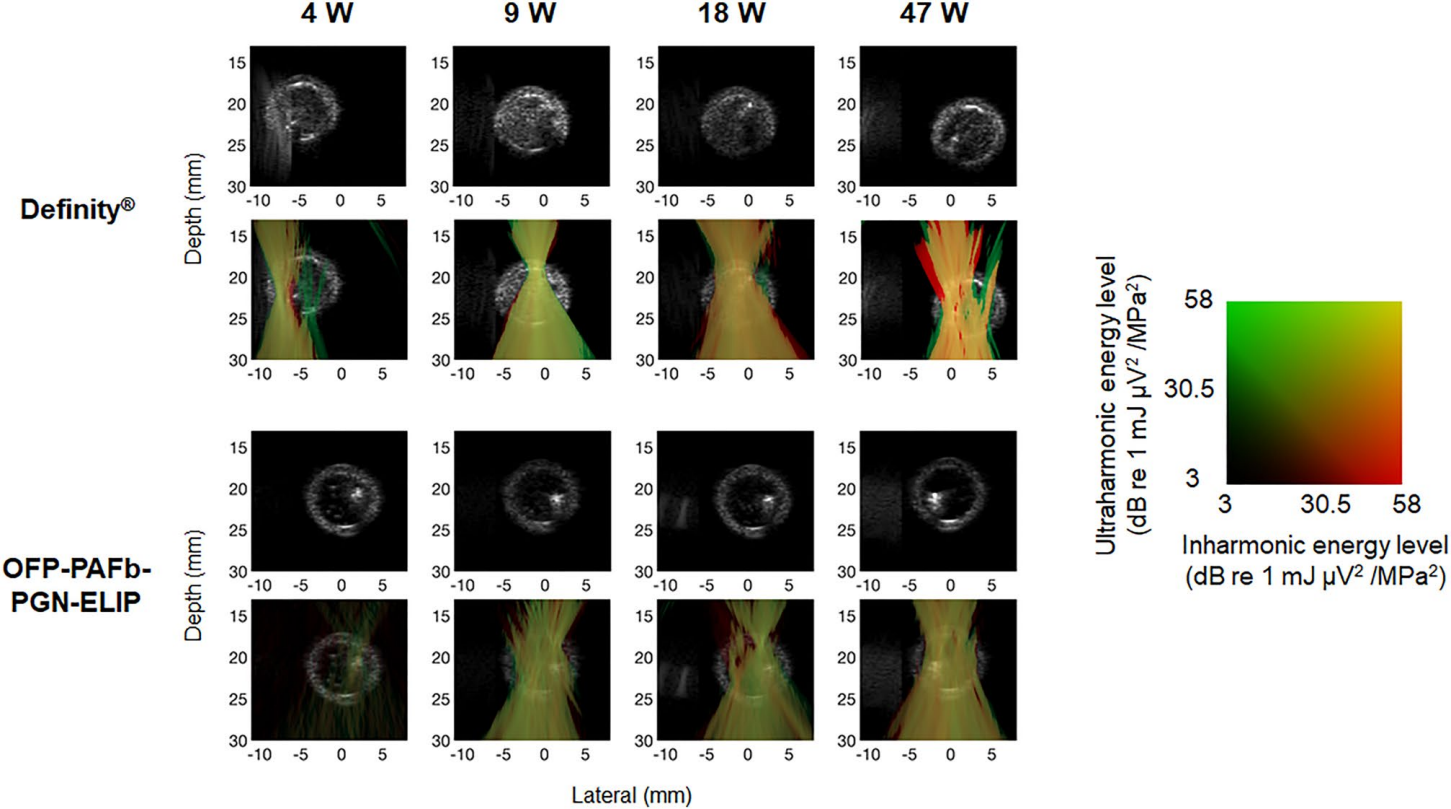
Composite PCI and B-Mode images of ultraharmonic (green) and inharmonic (red) emissions from cavitation nucleated by infused Definity or OFP-PAFb-PGN-ELIP insonified by one pair of EkoSonic transducers driven with 4, 9, 18, or 47 W electrical power. The B-mode images (visualizing echogenicity alone) are shown in rows 1 and 3, and the corresponding composite images are shown in rows 2 and 4.

Figure 6 displays the spatial distribution of microbubble echogenicity and cavitation energy within the tube lumen when either Definity or OFP-PAFb-PGN-ELIP were infused through EkoSonic catheters. The images were acquired when the L11-5v array was over the third active transducer pair in the EkoSonic catheter. Ultraharmonic and inharmonic emissions were observed at all electrical drive powers for both infused agents. Cavitation emissions nucleated by Definity were readily observed throughout the lumen at 18 W and 47 W drive powers and inharmonic emissions were prominent at 47 W for the 55 dB dynamic range used in these images. Cavitation emissions nucleated by OFP-PAFb-PGN-ELIP were observed throughout the lumen at 9, 18, and 47 W. Ultraharmonic and inharmonic cavitation emissions were visualized along the catheter treatment zone at all electrical drive powers during Definity or OFP-PAFb-PGN-ELIP infusions (see Supplemental Figs. S1-S2 online). When the L11-5v was aligned over each of the six active transducer pairs during pullback, cavitation energy levels in the composite PCI videos were maximized (i.e., the color overlays brightened). A higher amount of ultraharmonic and inharmonic cavitation emissions was observed from Definity rather than OFP-PAFb-PGN-ELIP infusions through the EkoSonic catheter.

### Hydrodynamic pressure within the EkoSonic catheter during echo contrast agent infusion

**Figure 7:**
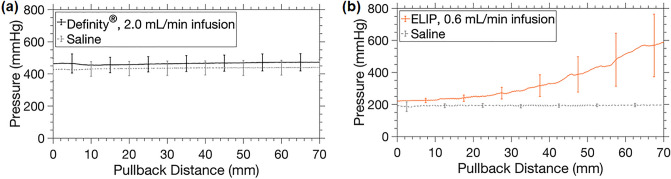
Hydrodynamic pressure within the EkoSonic catheter drug delivery lumen as (**a**) Definity microspheres or saline was infused at 2.0 mL/min or (**b**) OFP-PAFb-PGN-ELIP or saline was infused 0.6 mL/min, respectively. Error bars represent ± standard deviation, *n* = 12 each.

The hydrodynamic pressure within the EkoSonic catheter drug delivery lumen as Definity microspheres or OFP-PAFb-PGN-ELIP were infused is shown in Fig. 7. A time-averaged luminal hydrodynamic pressure of 468.5 ± 12.5 mmHg or 434.7 ± 5.6 mmHg (mean ± s.d., *n* = 12 each) was measured throughout the Definity or saline infusions, respectively, at 2.0 mL/min (Fig. 7a). At each individual point in time, the luminal hydrodynamic pressure during the Definity infusions was not statistically different than the saline infusions (multiple Kolmogorov–Smirnov tests, *n* = 12 per time point, alpha = 0.05, *p* > 0.05). A time-averaged pressure of 193.6 ± 3.4 mmHg was sustained in the catheter drug lumen throughout saline infusions at 0.6 mL/min (mean ± s.d., *n* = 12), but the pressure increased over the course of OFP-PAFb-PGN-ELIP infusions at 0.6 mL/min (Fig. 7b). At time points during the pullback, the saline and OFP-PAFb-PGN-ELIP luminal hydrodynamic pressures were statistically different (multiple Kolmogorov–Smirnov tests, *n* = 12 per time point, alpha = 0.05, *p* < 0.05).

## Discussion

Therapeutic-loaded microbubbles have been investigated for clinical applications^24^ including intravascular drug delivery^3,25^ and sonothrombolysis^8,11^. Previous characterization of the size distribution of therapeutic-loaded echogenic liposomes using the Coulter principle has yielded a range of particle sizes from 0.6 µm to 7 μm^3,8,9,11,25,26^. The minimum size that can be measured with this technique is 0.6 µm and smaller particles are possibly present. Peak number densities of therapeutic-loaded echogenic liposomes in the literature range from 2.6 × 10^6^ to 1.4 × 10^12^ particles per mL, depending on the formulation^3,8,9,11,25–27^. The size of the OFP-PAFb-PGN-ELIP in this study primarily ranged from 0.6 to 3.0 µm and the peak number density was 5.0 × 10^8^ echogenic liposomes per mL (Fig. 2a), which is consistent with previous measurements of therapeutic-loaded echogenic liposomes^3,8,9,11,26^. In the human adult, capillaries are 4 to 8 µm in diameter and red blood cells are 6 to 8 µm in diameter^28,29^. About 99% of OFP-PAFb-PGN-ELIP in this study were less than five microns in diameter (Fig. 2a) and would pass through capillaries readily. After intra-arterial infusions of Definity into rat muscles, microspheres greater than five microns in diameter were transiently (~ 10 min) trapped in the small arterioles and capillaries^30^. Because the size of OFP-PAFb-PGN-ELIP are slightly larger than Definity, future studies are needed to assess the passage of intra-arterial infusions of OFP-PAFb-PGN-ELIP through the capillary beds. Also needed are pharmacokinetic-pharmacodynamic preclinical studies to determine the biodistribution of the lipids, OFP, and PGN after infusion.

Attenuation of the OFP-PAFb-PGN-ELIP decreased after infusion through the EkoSonic catheter (Fig. 2b), which is consistent with the loss of Definity attenuation after infusion through the same catheter^22,23^. OFP-PAFb-PGN-ELIP agglomeration was observed after dilution and infusion, but only after several hours. This type of agglomeration was not observed for Definity. Thus, dilution might have affected the stability of the OFP-PAFb-PGN-ELIP during the attenuation measurements with the impulse method^26^. The exact amount of dilution during OFP-PAFb-PGN-ELIP bolus infusion and saline flush in the catheter was unknown but did not exceed 1:10 v/v. The decrease in both the number density and attenuation upon infusion (Fig. 2) was likely due to the retention or destruction of the OFP-PAFb-PGN-ELIP within the EkoSonic catheter. We hypothesize retention is either due to material interaction or to a size discrepancy between the OFP-PAFb-PGN-ELIP and the delivery holes of the catheter, which are 38 to 46 µm in diameter. Lafond et al.^22,23^ found that infusion rate impacted the size distribution of Definity infusing through the EkoSonic catheter. Talu et al.^31^ reported that lipid-encapsulated and perfluorocarbon-filled microbubbles were sensitive to orifice size and infusion rate. Decreasing orifice size and increasing infusion rates caused a decrease in microbubble number density and diameter^31^. Fouling of the EkoSonic catheter drug delivery holes with amphiphilic lipid or hydrophobic PGN was likely responsible for the increase in hydrodynamic pressure during each OFP-PAFb-PGN-ELIP infusion (Fig. 7b).

Future studies are needed to determine if the total PGN dose delivered after infusion through the EkoSonic catheter is sufficient for therapeutic effect. Klegerman et al*.*^9^ reported that each vial of OFP-PAFb-PGN-ELIP (also with a biotin-conjugated lipid to enable fluorescent imaging) contained 86.9 ± 11.9 µg PGN/mg lipid (mean ± s.d.). Each vial used in our study contained 5 mg lipid and 432.0 ± 128.3 µg PGN (Fig. 3), or 86.4 ± 14.2 µg PGN/mg lipid, which was consistent with the results of Klegerman et al.^9^, for a similar liposomal formulation. In a porcine model of peri-stent restenosis, Klegerman et al.^9^ infused nitroglycerine and dinitrophenyl-labeled PAFb-PGN-ELIP through the EkoSonic catheter and found that ultrasound exposure increased penetration of the dinitrophenyl-labeled PAFb-PGN-ELIP into arterial walls. Kee et al.^3^ also found that sequential infusion of nitric oxide (NO)-loaded echogenic liposomes and PGN-loaded echogenic liposomes conjugated with anti-intercellular adhesion molecule-1 (ICAM-1) antibody through the EkoSonic catheter was sufficient to prevent neointimal hyperplasia in stented arteries.

The acoustic output from all four drive electrical powers of the EkoSonic catheter was sufficient to sustain cavitation from infusions of Definity or OFP-PAFb-PGN-ELIP along the distal six transducer pairs of the catheter (Fig. 5). Note that our infusion strategy necessitated that the first six transducer pairs were quiescent to enable the echo contrast agents to be delivered into the tube lumen before ultrasound exposure^22^. The acoustic output of the EkoSonic catheter as a function of electrical drive power is provided in Table 1. As the drive power increased from 4 to 18 W, both ultraharmonic and inharmonic energy increased (Fig. 5). Above an 18 W drive power, the inharmonic energy also increased for Definity infusions. Note that only the ultraharmonic energy in excess of the inharmonic energy is plotted, thus providing an estimate for stable cavitation energy. The peak ultraharmonic and inharmonic cavitation energy nucleated by infused Definity during pullback at an electrical drive power of 9 W was on the order of 1 mJ µV^2^/MPa^2^ (Fig. 5). Note that the cavitation energy level calculated by Lafond et al.^22^ contained errors in the PCI processing algorithm and code^23^. The amount of cavitation energy sustained by infused Definity exceeded that of OFP-PAFb-PGN-ELIP by 1 to 2 orders of magnitude, likely due to the difference in attenuation and echogenicity of Definity (a blood pool contrast agent) versus drug-loaded echogenic liposomes (a theragnostic agent).

**Table 1:** Rarefactional pressure maximum and MI at the exterior surface of the EkoSonic catheter over each transducer pair. However, the assumptions about the conditions for cavitation embedded in the definition of the MI are not met^63^.

Electrical drive power (W)	4	9	18	47
Pressure (MPa)	0.45 ± 0.05	0.67 ± 0.07	0.95 ± 0.11	1.47 ± 0.16
MI	0.30 ± 0.03	0.45 ± 0.05	0.63 ± 0.07	0.98 ± 0.11

Surprisingly, both ultraharmonic and inharmonic emissions indicative of sustained stable and inertial cavitation^32^, respectively, are seen throughout the Definity infusions driven at 47 W, visualized as yellow in passive cavitation composite images and videos (Fig. 6, Supplemental Figs. S1-2 online). At the same output power, primarily stable cavitation was visualized during the OFP-PAFb-PGN-ELIP infusions, though some inertial cavitation was present as well. The amount of stable cavitation sustained by infused Definity at the 9, 18, and 47 W drive powers was equivalent, though the inertial cavitation increased with drive power (Figs. 5, 6). Concerning the spatial distribution of cavitation activity, as power increased cavitation emissions were detected in a larger percentage of the tube lumen for both agents. Definity infusions nucleated cavitation emissions throughout the lumen over the entire 3 min infusion (Supplementary Fig. S1 online). Mapping bubble activity has the potential to inform the relationship between cavitation (stable and inertial) and cellular response. The specific contribution of stable and inertial cavitation to enhanced therapeutic delivery beyond the endothelium is unknown at this time.

Cavitation has been shown to mediate drug delivery across the blood–brain barrier^33^, and enhance drug delivery to vascular tissue^25,34^, tumors^35^, and biofilm^36^. The mechanical interaction of acoustically active microbubbles and endothelial cells has been studied extensively by Beekers et al.^14,37–39^. Beekers et al. observed different degrees of tight junction opening depending on the radial excursion of individual microbubbles in the vicinity of endothelial cells. Belcik et al.^40^ showed that cavitation-induced flow augmentation in mice was mediated by shear-dependent release of adenosine triphosphate (ATP) from the endothelium and erythrocytes, and subsequent production of NO, prostaglandins, and adenosine. Muller et al. also observed ultrasound-triggered ATP and NO release from erythrocytes exposed to the EkoSonic catheter aligned over the murine femoral artery^41^. The production of NO initiates paracellular transport beyond endothelial cells lining the vasculature^34^, a key to ultrasound-enhanced drug delivery. Therefore, sustaining cavitation throughout the lumen with ultrasound exposure from the EkoSonic catheter, noted in our study at drive powers above 9 W (Fig. 6), will likely enhance PGN delivery. Indeed, both Kee et al.^3^ and Klegerman et al.^9^ demonstrated enhanced delivery to stented arterial tissue infused with targeted, PGN-loaded ELIP and exposed to the EkoSonic catheter driven with a 9 W electrical drive output (0.62 MPa peak rarefactional pressure, Table 1) in an atherosclerotic miniature swine model. However, the specific cavitation energy and type required to enhance the pioglitazone uptake and avoid deleterious effects is unknown at this time. More studies are needed to determine the long-term safety of ultrasound-mediated pioglitazone delivery in arterial tissue.

A limitation of the present work was that the frequency-dependent sensitivity of the PCI array was not calibrated^42^, which would enable comparison of cavitation activity from other laboratories quantitatively. Quantitative measurement of absolute energy emissions can be used to monitor cavitation-mediated bioeffects spatially^43,44^ and temporally^45,46^. The use of PCI coupled with an assessment of neointimal hyperplasia after pioglitazone delivery would allow the choice of acoustic parameters to be optimized to obtain this beneficial bioeffect. Recent publications suggest that both inertial cavitation and stable cavitation promote drug delivery^47–50^. Inertial cavitation opens endothelial tight junctions^12–14^, and acoustic streaming caused by stable or inertial cavitation^51–54^ increases drug transport across the endothelium^16,17^. The poor axial resolution in PCI with linear arrays using the delay, sum, and integrate beamforming technique^22,23,32^ may limit the image guidance capability. Further development of strategies to improve the spatial resolution and accuracy of PCI using phase coherence factor^55,56^ or robust Capon beamforming^57^ are needed.

The EkoSonic catheter described in this work was cleared by the FDA for the delivery of physician specified fluids, including thrombolytics, into the peripheral vasculature. Our data supports an emerging application of ultrasound-mediated therapeutic delivery via a liposomal formulation that includes octafluoropropane gas which nucleated bubble activity during 3 min infusions. Importantly, the OFP-PAFb-PGN-ELIP infused through the EkoSonic catheter produced a scant amount of inertial cavitation and promoted stable cavitation, potentially clearing the way for first in human studies. This catheter-based intra-arterial drug delivery strategy has the potential to stabilize atheroma in the peri-stent region, preventing neointimal atherogenesis at the time of intervention. Further studies are needed to correlate the amount and type of cavitation with drug penetration into vascular tissue with therapeutic effect.

## Methods

### EkoSonic catheter

**Figure 8:**
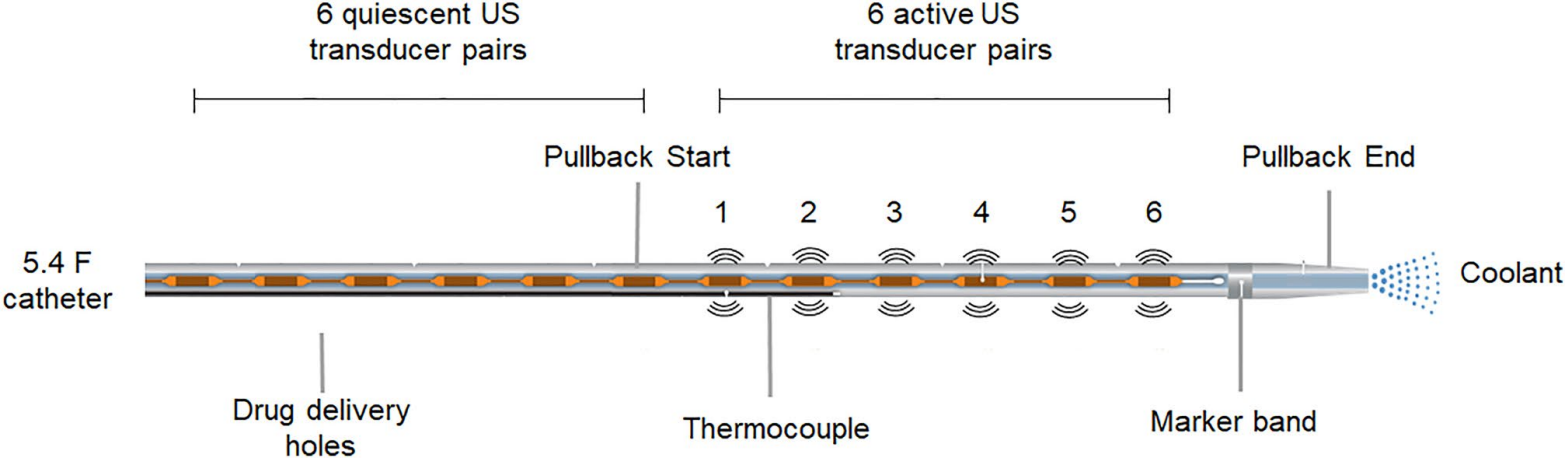
Schematic of EkoSonic catheter drug delivery hole and transducer layout. Along the 12 cm treatment zone of the catheter, the first six ultrasound transducer pairs were quiescent while the distal six transducer pairs were active and insonified the echogenic liposomes. The first drug delivery hole was located 0.5 cm after the first of six quiescent transducer pairs (modified from Lafond et al.^22^).

The 5.4 F (1.8 mm diameter) EkoSonic catheter used in this study had a 12 cm treatment zone with 12 pairs of 2 mm long US transducers located within the catheter. Transducer pairs were spaced 10 mm apart, and 38 to 46 µm diameter drug delivery ports were located 5 mm distally to each transducer pair (Fig. 8). A programmable unit provided by Boston Scientific was used to drive the 2.25 MHz center frequency transducer pairs with 15 ms pulses at a 10 Hz pulse repetition frequency. The luminal hydrodynamic pressure within the EkoSonic catheter drug lumen was measured using an in-line sensor (PRESS-S-000, PendoTECH, Princeton, NJ, USA) over the course of Definity or OFP-PAFb-PGN-ELIP infusions.

### Definity Preparation

Definity (Lantheus, Billerica, MA, USA) was prepared according to the package insert. Each Definity vial (9.2 × 10^9^ microbubbles/mL) was kept at room temperature for one hour, activated, and used within ten hours of activation. If not used within five minutes, the vial was gently inverted repeatedly for 10 s to resuspend the microbubbles. For cavitation measurements, vials were vented to OFP, and Definity was withdrawn using an 18 G needle connected to a gastight 250 µL syringe (Hamilton Co., Reno, NV, USA). The Definity was transferred to a 10 mL syringe (Becton Dickinson, Franklin Lakes, NJ, USA) containing room-temperature, 0.9% NaCl saline so that the solution before infusion had a concentration of 2.33 × 10^8^ microbubbles/mL. This Definity concentration mimics the intravenous infusion dose recommended by the manufacturer’s package insert.

### Preparation of pioglitazone-loaded echogenic liposomes

Echogenic liposomes were manufactured according to a protocol developed by Klegerman et al.^9^ without the inclusion of a biotin-conjugated lipid to enable fluorescent imaging. Three phospholipids, 1,2-distearoyl-sn-glycero-3-phosphocholine (DSPC, Avanti Polar Lipids, AL, USA), 1,2-dioleoyl-sn-glycero-3-phosphoethanolamine-N-[4-(p-maleimidophenyl)butyramide] (MPB-DOPE, Avanti Polar Lipids, Alabaster, AL, USA), 1,2-dioleoyl-sn-glycero-3-phosphocholine (DOPC, Avanti Polar Lipids, Alabaster, AL, USA), and cholesterol (Avanti Polar Lipids, Alabaster, AL, USA) at a molar ratio of 52:8:30:10 were mixed. One-hundred milligrams of the lipid mixture and 10 mg PGN (Cayman Chemical, Ann Arbor, MI, USA) were dissolved in 20 mL absolute ethanol (Fisher Scientific, Hampton, NH, USA) and heated to 90 °C to improve the miscibility of the phospholipid and PGN into the ethanol solvent. The ethanolic solution was loaded into a 20 mL glass syringe with a 27 G needle and injected into 115 mL of 0.2 µm-filtered water (Milli-Q Advantage A10, Millipore Sigma, St. Louis, MO, USA) that was autoclaved (Primus, Omaha, NE, USA) and stirred at 1,000 rpm (Fisher Scientific, Hampton, NH, USA). Spontaneous formation of unilamellar liposomes occurred as soon as the organic phase was in contact with the aqueous phase. The liposomal dispersion was stirred for 5 min at room temperature. The final ethanol concentration in the liposomal dispersion was 14.8% v/v. The residual solvent was removed by rotary evaporation (RII, Buchi, Cornaredo, Italy). After the residual solvent was removed, the liposomes were resuspended with 0.32 M mannitol to produce a liposome concentration of 1 mg PGN/10 mg lipids/mL.

### Conjugation of PAFb fibrin-binding peptide to PGN-ELIP

For each 60 mg of PGN-loaded ELIP lipid, 1 mg of a thiolated fibrin-binding peptide was added in 1 mL of pH 6.7 citrate–phosphate buffer. A custom-made, nonapeptide, PAFb, H-Gly-Pro-Arg-Pro-Pro-Gly-Gly-Gly-Cys-NH_2_ HCl (GPRPPGGGC), contains the pentapeptide GPRPP^58,59^ which binds to fibrin, a marker for late-stage atheroma^60,61^. Conjugation was achieved with a thioether linkage between the lipid, MPB-DOPE, and the carboxy-terminal peptide cysteinyl thiol group (separated from the fibrin-binding moiety by a tri-glycyl spacer). The pH was adjusted to 6.5–6.7 with 1.0 M sodium hydroxide, the mixture was topped with argon, and incubated with stirring at 180–190 rpm overnight at room temperature in the dark.

After the reaction, the mixture was made isotonic with 6% sodium chloride and centrifuged in 1.5 mL polypropylene tubes in a microfuge (Eppendorf miniSpin plus, Eppendorf, Hamburg, Germany) at 10,000 rpm for 10 min at room temperature. Supernates were discarded and pellets washed twice centrifugally with 1.0 mL 0.02 M phosphate-buffered saline, pH 7.4, per tube. Pellets were resuspended to greater than 10 mg lipid/mL with 0.32 M D-mannitol and pooled. Lipid recovery relative to the reaction mixture was determined by optical absorption at 280 nm (A_280_) and the pooled pellet was brought to 10 mg lipid/mL with 0.32 M D-mannitol. Aliquots of this mixture (0.5 mL) were distributed into 3 mL crimp-top vials (Wheaton, Sigma-Aldrich, St. Louis, MO, USA), frozen for ≥ 2 h at -80 °C, and lyophilized (FreeZone 6-L, Kansas City, MO, USA) for 48 h. Vials were capped with rubber inserts, sealed with aluminum crimp caps, and charged with octafluoropropane (OFP) at 1 atm pressure prior to evaluation and use. The OFP-PAFb-PGN-ELIP were shipped overnight to the University of Cincinnati with refrigerant packs and used for experiments within 8.5 mos.

### OFP-PAFb-PGN-ELIP reconstitution

Prior to reconstitution, each vial of OFP-PAFb-PGN-ELIP was left at room temperature for three hours then vented to room air using a 22 G needle. Using an 18 G needle, 0.5 mL of room-temperature, air-saturated, 0.2 micron-filtered water (Nanopure, Barnstead Thermolyne, Dubuque, IA, USA) was slowly added to the vial. The vial was gently swirled by hand until the OFP-PAFb-PGN-ELIP resuspended completely. The reconstituted OFP-PAFb-PGN-ELIP were used within 15 min.

### Catheter infusion protocols for Definity and OFP-PAFb-PGN-ELIP

Each EkoSonic catheter was primed with saline. Definity was infused into the drug port of the EkoSonic catheter using a syringe pump (Legato 180, KD Scientific, Holliston, MA, USA) at 2.0 mL/min. The rate of 2.0 mL/min has been shown to minimize the effects of flow rate on Definity size distribution and acoustic attenuation when infused through an EkoSonic catheter^22^. The 0.5 mL OFP-PAFb-PGN-ELIP volume was infused using the same syringe pump at 0.6 mL/min, the infusion rate used by Klegerman et al. in a porcine model of peri-stent restenosis^9^. When the OFP-PAFb-PGN-ELIP infusion was completed, 2.5 mL of room temperature, air saturated saline was infused at 0.6 mL/min to push the OFP-PAFb-PGN-ELIP through EkoSonic catheter drug ports.

### OFP-PAFb-PGN-ELIP size distribution measurements

A Multisizer 4 particle size analyzer (Beckman Coulter, Indianapolis, IN, USA) was used to measure the size distribution of OFP-PAFb-PGN-ELIP with and without infusion through the EkoSonic catheter. Each EkoSonic catheter was used to infuse a single OFP-PAFb-PGN-ELIP vial without driving the ultrasound transducer pairs^22^. During the infusion through the catheter, 3 mL of effluent was collected. After the infusion, 17 mL of saline was added to the effluent and 0.02 mL of the 20 mL effluent solution was added to 9.98 mL saline. Aliquots taken directly from the reconstituted OFP-PAFb-PGN-ELIP vial were serially diluted in saline to achieve a 1:20 × 10^3^ v/v dilution, similar to the dilution of the infused aliquots. The size distribution was measured using a 30 µm aperture. The particle size distribution in saline was also measured and subtracted from the OFP-PAFb-PGN-ELIP size measurements. Size distribution mean and standard deviation values (corrected for dilution) were assessed using GraphPad Prism (version 9.2.0, San Diego, California USA).

### Acoustic attenuation spectroscopy

An acoustic attenuation spectroscopy system^26^ was used to determine the attenuation coefficient of OFP-PAFb-PGN-ELIP from 2 to 25 MHz directly from the vial or after infusion through EkoSonic catheters. The attenuation frequency range of 2 to 25 MHz corresponds to the -20-dB bandwidth of the system. Each EkoSonic catheter was used to infuse a single OFP-PAFb-PGN-ELIP vial without acoustic activation of the transducer pairs and thereafter discarded. During the infusion through the catheter, 3 mL of effluent was collected. After the infusion, 17 mL of saline was added to the effluent. Aliquots taken for measurements directly from the reconstituted OFP-PAFb-PGN-ELIP vials were serially diluted in saline to reach a 1:40 v/v dilution, similar to the dilution of the infused aliquots. OFP-PAFb-PGN-ELIP samples flowed into the sample chamber (CLINI-cell, Mabio, Tourcoing, France) by gravity, and a broadband substitution technique was used to determine the frequency-dependent attenuation coefficient of each sample, in decibels per centimeter^26^. The mean and standard deviation attenuation at 2.2 MHz were assessed using GraphPad Prism (version 9.2.0).

### Measurement of infused pioglitazone dose

A PGN quality control standard in 80% ethanol was run through an HPLC system consisting of a 6 × 300 mm YMC ODS-A 5 µm C18 column (Waters, Milford, MA, USA), a dedicated PC running Empower 2 software, a 2996 Photodiode Array Detector, a 717 Plus Autosampler, and a Delta 600 Controller. The mobile phase was 60% acetonitrile and 40% methanol, the injection volume was 20 µL, the detection wavelength was 269 nm, and the flow rate was 1 mL per minute. OFP-PAFb-PGN-ELIP samples at an 1:40 v/v dilution were prepared either directly from the vial or infused through a quiescent EkoSonic catheter. Samples were further diluted 1:5 or 1:10 v/v in 80% ethanol and run through the HPLC system. Two HPLC duplicates were made per sample dilution (*n* = 4 per vial) and PGN dose was calculated from the slope of a composite standard curve, and adjusted for the PGN quality control standard, as AUC/(µg PGN/mL). Repeated measure, linear mixed model, Type II Wald Chi-Square tests were carried out in R (version 4.2.1, R Foundation for Statistical Computing, Vienna, Austria) to compare the PGN measurements directly from the vial and after infusion through EkoSonic catheters.

### Spatial light interference microscopy of contrast agents

Quantitative phase imaging (QPI)^62^ relies on the principle of interference, where phase differences between a sample and a reference field are measured experimentally. Using this technique, transparent objects such as live cells and lipid-based microbubbles are imaged with high contrast and sensitivity using the phase information of the field^62^. An optical module (PHI OPTICS INC., Champaign, IL, USA) was added to the output port of a phase contrast microscope (Axio Observer 7, Carl Zeiss Microscopy LLC, White Plains, NY) with a 10 × objective (Objective EC "Plan-Neofluar" 10x/0.30 M27, Carl Zeiss Microscopy). For each image acquisition, Definity or OFP-PAFb-PGN-ELIP at a 1:1 × 10^4^ v/v dilution were immediately transferred to a 35 mm glass bottom dish with a 20 mm micro-well (Cellvis LLC, Mountain View, CA). An 18 mm diameter #0 coverslip was placed on top of the droplet sample to minimize the sample meniscus.

### Physiologic flow model

**Figure 9:**
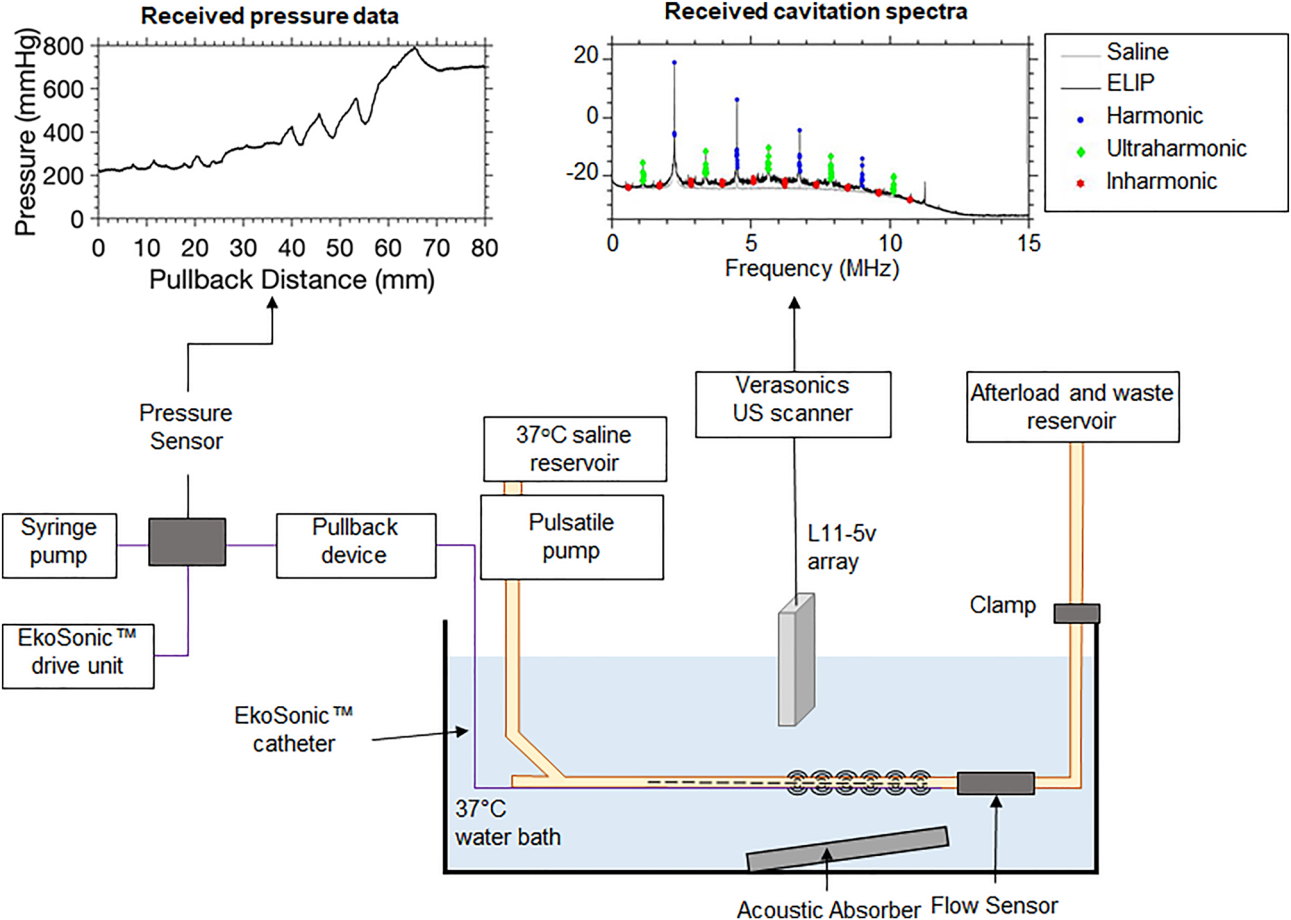
Schematic of flow phantom setup for passive cavitation imaging. Saline at 37 °C was pumped from the reservoir, over the catheter, to the afterload, and to the waste reservoir. During each experimental run, the catheter was pulled back within the tubing so that the L11-5v array scanned across the active transducer pairs (modified from Lafond et al.^22^).

A model of porcine femoral arterial flow (Fig. 9) was adapted from Lafond et al.^22^. Saline held in a 37 °C reservoir (BW-20B, Lab Companion, Yuseong-gu, Daejeon, Republic of Korea) was pumped through 6.35 mm-inner diameter latex tubing with a 0.79-mm wall thickness using a pulsatile pump (Model 1407, Harvard Apparatus, Holliston, MA, USA) with a time-averaged volumetric flow rate of 100 mL/min. A flow sensor (ME6PXN, Transonic, Ithaca, NY, USA) connected to a flow module (TS410, Transonic) enabled confirmation of the 100 mL/min time-averaged flow rate before echo contrast agent infusion, which disrupted the function of the flow sensor. The flow module was connected to a data acquisition (DAQ) board (PL3508 PowerLab 8/35, ADInstruments, Bella Vista NSW 2153, Australia). Hydrodynamic pressure was measured using an in-line sensor (PRESS-S-000, PendoTECH, Princeton, NJ, USA) over the course of the infusion of Definity or OFP-PAFb-PGN-ELIP. The pressure sensor was connected to the DAQ board via an OMEGA bridge sensor (DMD-475, OMEGA Engineering, Inc., Stamford, CT, USA). LabChart software (version 8.1.11, ADInstruments, Bella Vista NSW 2153, Australia) was used to record volumetric flow and hydrodynamic pressure data, and MATLAB (R2018b, The MathWorks Inc., Natick, MA, USA) was used to plot the data. An L11-5v linear array (Verasonics, Kirkland, WA, USA) with a transverse view of the latex tube was connected to a Vantage 256 US scanner (Verasonics) to obtain both B-mode images and PCI data^32^.

The L11-5v linear array was positioned so that the tube lumen center was 4 mm beyond the natural focus of the array (18 mm). The location and temporal extent of ultraharmonic and inharmonic cavitation was acquired for Definity or OFP-PAFb-PGN-ELIP infused through the EkoSonic catheter, driven at 4, 9, 18, or 47 W pulse average electrical power. The FDA-approved clinical pulsed ultrasound protocol utilized by the EkoSonic catheter varies between 9 and 47 W. Table 1 provides the peak rarefaction pressure field measurements at the surface of the EkoSonic catheter as a function of electrical drive power, as well as the mechanical index, MI. Note however, that the assumptions about the conditions for cavitation embedded in the definition of the MI are not met^63^. A pulse duration of 15 ms exceeds the single cycle assumption needed to neglect rectified diffusion^63^. To enable contrast agent infusion from the first six drug delivery ports before ultrasound exposure, only the distal six transducer pairs were acoustically activated. PCI data were acquired over the distal six transducer pairs, as the six distal pairs were used by Kee et al.^3^ to deliver PGN-loaded ELIP targeted to ICAM-1 to prevent peri-stent neointimal atherogenesis in a porcine model of atherosclerotic arterial disease.

The EkoSonic catheter was primed with room temperature saline, attached to a Volcano R100 pullback device (Philips, Koninklijke, NV, USA), and inserted into the flow tubing using a hemostasis valve. The catheter was aligned so that the L11-5v array was positioned 10 mm before the distal six transducer pairs. Definity or OFP-PAFb-PGN-ELIP was infused, and data was acquired (see Supplementary Fig. S5 online). Data analysis was completed within MATLAB (R2018b) and GraphPad Prism (version 9.2.0). The distribution of the luminal hydrodynamic pressure over time during Definity and saline infusions (2 mL/min) were assessed for normality using Kolmogorov–Smirnov test. Then multiple Kolmogorov–Smirnov tests with a two-stage method of Benjamini, Krieger, and Yekutieli^64^ were used to compare the luminal hydrodynamic pressures at each time point. The same statistical methods were used to compare the OFP-PAFb-PGN-ELIP and saline (0.6 mL/min) luminal hydrodynamic pressure data.

### Passive cavitation imaging

Cavitation activity was assessed by acquiring B-Mode and PCI data during Definity or OFP-PAFb-PGN-ELIP infusions through the EkoSonic catheter. A pullback device was used to move the catheter through the PCI imaging plane between the quiescent sixth transducer pair and 20 mm beyond the most distal active transducer pair (Fig. 8). A pullback rate of 0.5 mm/s was used for Definity infusions and a pullback rate of 1 mm/s was used for OFP-PAFb-PGN-ELIP infusions to capture the duration of cavitation spawned by both schemes. Acoustic emissions were recorded every 1.0 ± 0.2 s (mean ± s.d.). Acoustic emission spectra, including inharmonics, subharmonics, and ultraharmonics were independently beamformed^22,23^ on a personal computer (Dell Precision 5820, Round Rock, TX, USA) using custom MATLAB code (R2018b). Cavitation data from saline infusions served as a baseline and were subtracted from the Definity and OFP-PAFb-PGN-ELIP cavitation data.

PCI beamforming, cavitation energy calculations, and composite image formulations were adapted from Lafond et al.^22^with corrections in the beamforming algorithm and code^23^. Data acquisition commenced immediately after the EkoSonic transducer pairs were triggered. Acquired datasets were divided into 288 µs windows to minimize spectral leakage^65^, and the second 288 µs window of the non-beamformed signal was processed to avoid the initial transient^22^. Beamforming was performed in the Fourier domain, integrating the energy over 40 kHz bands centered about the subharmonic and ultraharmonic frequencies between 4.68 and 10.52 MHz. The discretized signal cavitation energy was calculated using Eq. 1 in Lafond et al.^22^ with corrections^23^. For each pulse, ultraharmonic and inharmonic emission images were beamformed according to Eq. 2 in Lafond et al.^22^ with negative energy values set to zero in order to take advantage of frequency compounding speckle reduction^66^. The maximum cavitation energy level in the composite PCI images was set to the maximum inharmonic emissions from Definity infusions, and the dynamic range was set to 55 dB re 1 mJ µV^2^/MPa^2^ to encompass the minimum emissions from OFP-PAFb-PGN-ELIP infusions. Total cavitation energy was computed according to Eq. 8 in Lafond et al^22,23^ with negative energy values also forced to zero.

## Supplementary Information

Supplementary Video 1.

Supplementary Video 2.

Supplementary Video 3.

Supplementary Video 4.

Supplementary Information 1.

Supplementary Information 2.

## Data Availability

Datasets are available from the corresponding author on reasonable request.
