# Supplementary figures and images for: Initiating and imaging cavitation from infused echo contrast agents through the EkoSonic catheter
Source: Sci Rep. 2023 Apr 16;13:6191. doi: 10.1038/s41598-023-33164-5 (PMC10106464; doi:10.1038/s41598-023-33164-5)

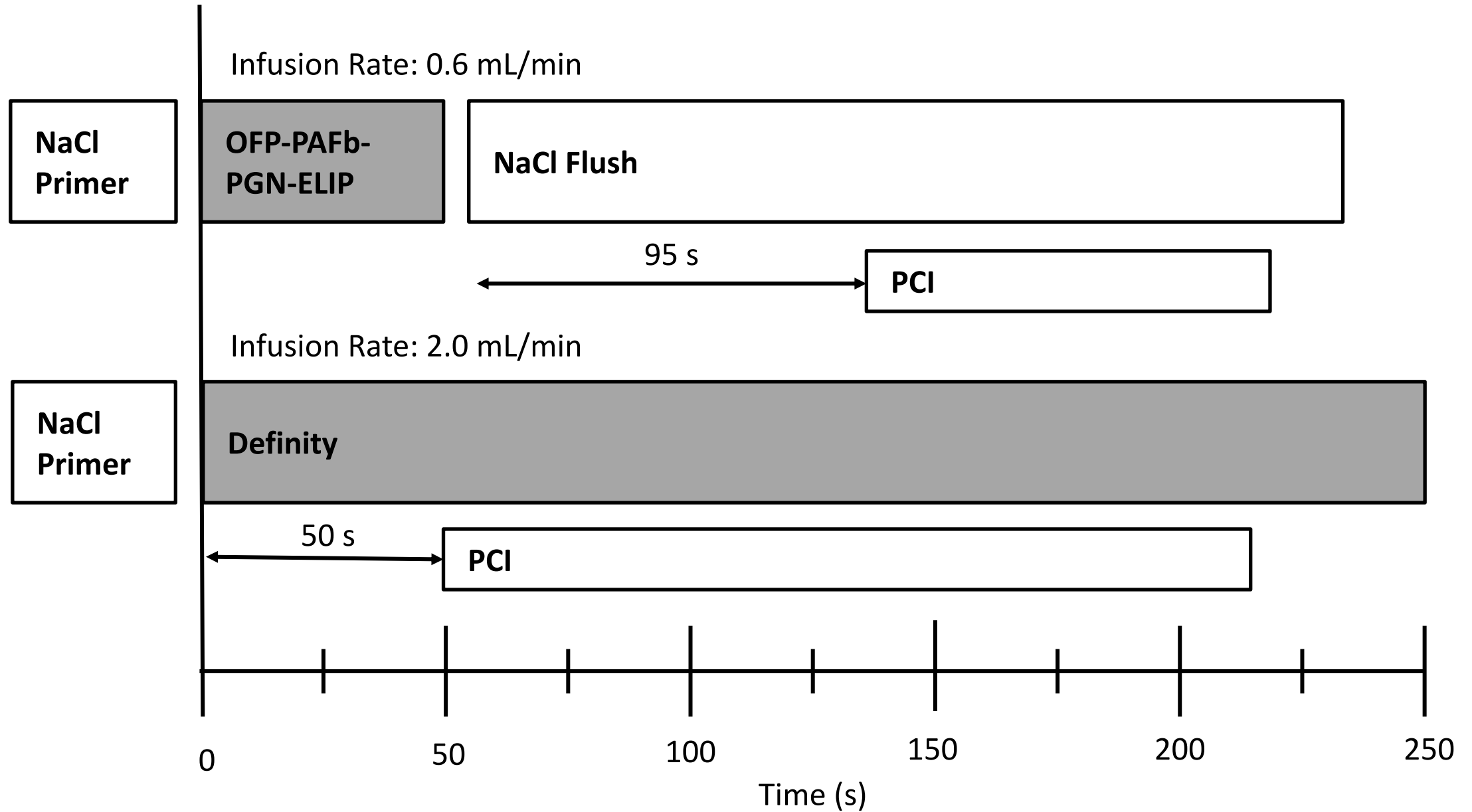

Supplement: Supplementary file 5 — Supplementary Information 1. [file 41598_2023_33164_MOESM5_ESM.pdf]
