# Supplementary material for: Initiating and imaging cavitation from infused echo contrast agents through the EkoSonic catheter
Source: Sci Rep. 2023 Apr 16;13:6191. doi: 10.1038/s41598-023-33164-5 (PMC10106464; doi:10.1038/s41598-023-33164-5)
Supplement: Supplementary file 6 — Supplementary Information 2. [file 41598_2023_33164_MOESM6_ESM.docx]

**List of Supplementary Figure Legends**

**Supplementary Figure S1.** Composite PCI videos of ultraharmonic (green) and inharmonic (red) emissions from cavitation nucleated by infused Definity insonified by one pair of EkoSonic transducers driven with 4, 9, 18, or 47 W electrical power. The B-mode only images (to visualize echogenicity alone) are shown in row 1, with the corresponding composite images in row 2. A pullback rate of 0.5 mm/s was used for Definity infusions to capture the duration of cavitation.

**Supplementary Figure S2.** Composite PCI videos of ultraharmonic (green) and inharmonic (red) emissions from cavitation nucleated by infused OFP-PAFb-PGN-ELIP insonified by one pair of EkoSonic transducers driven with 4, 9, 18, or 47 W electrical power. The B-mode only images (to visualize echogenicity alone) are shown in row 1, with the corresponding composite images in row 2. A pullback rate of 1 mm/s was used for OFP-PAFb-PGN-ELIP infusions to capture the duration of cavitation.

**Supplementary Figure S3.** Spatial light interference microscopy (SLIM) videos of a field of Definity microspheres at 10x magnification. Images were taken of microspheres withdrawn directly from vials (no infusion).

**Supplementary Figure S4.** Spatial light interference microscopy (SLIM) videos of a field of OFP-PAFb-PGN-ELIP at 10x magnification. Images were taken of OFP-PAFb-PGN-ELIP withdrawn directly from vials (no infusion).

**Supplementary Figure S5.** Diagram of infusion timing for passive cavitation measurements. PCI acquisition occurred while Definity or OFP-PAFb-PGN-ELIP was infused through the EkoSonic catheter into the tube lumen and the catheter was pulled through the beam of the L11-5v transducer array.
